# Remarks on the Effects of Iodine on the Glandular System, and on the Properties of Kousso

**Published:** 1852-10

**Authors:** Thomas H. Silvester

**Affiliations:** Clapham


### MATERIA MEDICA AND THERAPEUTICS.

*Remarks on the Effects of Iodine on the Glandular System, and on the Properties of Kousso.* By THOMAS H. SILVESTER, M. D., Clapham.

[Read at the Anniversary Meeting of the South-Eastern Branch.]

### ON THE EFFECTS OF IODINE ON THE GLANDULAR SYSTEM.

In our journal the question has been asked,—Whether atrophy or absorption ever takes place in the glandular system from the use of iodine? In answer to this question, I would beg the favor of the present members of the Society to allow me to make a few remarks, the result of many years' attention to this point. From 1834 to 1844 a great many patients, suffering under secondary or tertiary syphilis, were admitted into St. Thomas' Hospital, more especially under the care of the late Dr. Williams, who had gained a high reputation in the treatment of these morbid symptoms. Most of these patients came under my notice and particular observation, and many of the remarkable cases were entered in my note-book, but not one instance of atrophy or absorption of the large glands, occurred in our experience. It was thought advisable, on the recommendation of Lugol, to test the efficacy of the iodide of potassium in scrofulous enlargement of the glands, and in order to give M. Lugol's method of treatment fair play, a most characteristic specimen of these affections was selected. A young woman, fat, florid, and fair, aged 18, was admitted with suppurating glands at the angle of the jaw, and others approaching suppuration, or hard and inflamed, extending to the chin, were conspicuously prominent. Eight grains of the iodide of potassium, in camphor mixture, were prescribed, and steadily administered, for nearly six months, without the slightest perceptible effect upon the scrofulous mass of glands, and she was presented in much the same state as at her admission. Now, it happened that in this girl the breasts were largely developed, but no change was produced in their size by the treatment adopted for the scrofulous ailment, notwithstanding the full dose, and prolonged administration of the iodide.

There were at this period, before the treatment had become generally known, innumerable cases of syphilitic periostitis, in which the iodide of potassium was very successful, and yet we never witnessed atrophy or absorption of either the breast or testicle during the use of this remedy. A case of simple hypertrophy of the breasts was then made the subject of experiment; eight grains of the drug were taken, steadily and continuously, for three months, but no diminution of the mammæ took place.

A boy, aged 12, presented himself with immensely enlarged tonsils, and took the iodide nearly six months, without any impression having been made upon these organs. It would weary you to bring forward further illustration on this subject, and this negative kind of argument is, I am aware, not perfectly satisfactory, and may be destroyed by a single example of the positive power of the remedy in causing absorption of either the breast or testicle; but ten years' observation in a large hospital, failed to furnish me with a single proof in favor of the opinion that atrophy or absorption of the glandular system, in its normal condition, arises from the use of iodine in any form. Experience as to the topical application of this powerful agent, involves an inquiry into the effects of friction, stimulation, protection, and warmth, and excludes all inference as to its specific property. It must be confessed, that enlarged testicles not unfrequently yield to its influence, but it will be found, on inquiry, that in these cases the system has been contaminated by the syphilitic poison. The same remark is applicable to chronic induration of the inguinal glands. It is a very remarkable fact, that the swelling of the thyroid body, in common bronchocele, vanishes under the internal use of iodine, especially the iodide of potassium. The rapidity and certainty of its removal are equalled only by that of the venereal node; and I have sometimes thought that there may be a vital elective attraction between the iodine and the lime, which latter forms the basis of the nodal tumor, and is, probably, the chief element in the thyroid enlargement.

It still remains to be explained, how it happens that tumors, enlargement and thickenings of a nature other than have been noticed, disappear under the use, topical or internal, of the remedy in question: the explanation is undoubtedly difficult; but I may be allowed to remark, that there is an absence of permanency in the glands generally, the thyroid disappears spontaneously, the tonsils naturally at puberty, the breasts in advanced age, and sometimes the testicles and ovaries; and there are few practitioners who have not met with cases of absorption of the breasts and testicles from some unknown cause, and in morbid instances, when no medicine has been taken. I have over and over again known and seen large swellings vanish under the long-continued application of a poultice, or wet lint and oil silk; and an equal number of failures, where iodine, internally and externally, was had recourse to, have occurred to me.

I recommend this subject to my medical brethren, and I do not hesitate to say, that they will confer a great boon upon scientific medicine by determining, with certainty, the value and effect of iodine in the cure of disease.

### ON THE PROPERTIES OF KOUSSO.

Much difference of opinion still exists with regard to the specific property of kousso, or the Brayera anthelmintica. It has been lauded by some as universally efficacious in the treatment of tape-worm, whilst by others it is described as inferior to either turpentine or the preparations of male fern. My attention has been drawn to this subject, more particularly of late, from learning that the price of the drug has undergone considerable diminution, and that we are likely to have a regular, abundant, and cheap supply from Abyssinia. My first patient gave *one guinea* for two doses of half an ounce each. The traveller, Mons. Rochet d'Hericourt, charged M. Simon, his agent in London, £1 15s. per ounce. He had in his possession 1400 pounds, which, at even £1 10s. per ounce, would have produced him the enormous sum of 22,400 guineas. The ordinary price of the bruised flowers and leaflets, of which I have brought a specimen for your inspection, is two shillings the ounce. It is prudent to order the drug in this form, rather than as a powder, adulteration in the former case being more difficult of accomplishment.

I have not hesitated to prescribe the kousso, at its present reduced cost, even to my dispensary patients, and the cases about to be related, encourage me to hope that much benefit will be conferred upon the poor by its employment, (for it is they, chiefly, who suffer from the disorder,) and that ultimately it will be a recognised article of the materia medica. I now beg to read a case or two by way of illustration.

*Case* 1.—A girl, aged 15, came under my care at the Clapham General Dispensary, for worms; she had been suffering from this cause nearly six years, and had undergone a variety of treatment, which had been partially efficacious in having brought away long portions of tænia; but the head could never be discovered in the evacuations. It often happened that, after taking a strong purgative, she passed fifteen or twenty feet of her internal enemy, and yet long pieces were evacuated every week or ten days. Her night dress frequently contained some of the articulations, and she was occasionally obliged to leave school for the purpose of having extruding portions removed.

Having taken large doses of the spirit of turpentine in gruel, many times, and five grains of the sulphate of iron, with a drachm of salts, three times a day for several months, without avail, she discontinued her visits to the dispensary, and placed herself under the care of my friend Mr. Angers, as a private patient. It was then determined to administer the kousso, and a dose was procured at considerable expense—namely, one guinea. It proved entirely efficacious; an immense mass of the worm came away, in which, after a most careful and minute examination, the head was discovered. This part might easily escape observation, owing to its minuteness; it appears singularly disproportionate to the size and length of the animal, not being much larger than a pin's head. It is characterised by four black points like eyes, but which are really suckers, by the aid of which this parasite worm absorbs the chyle contained in the intestinal canal. I preserved this part of the animal for many months, in spirit of wine, but unfortunately my servant, by mistake, threw it away, to my very great annoyance. The first dose given to this patient answered the purpose, but it was thought advisable to repeat it, and accordingly another half ounce of the powder was infused in hot water, and taken the day after a gentle purge of castor oil. The whole of the worm seemed to have been removed, for the kousso acted gently, as before, upon the bowels, but no articulations were found; the patient remained free from further inconvenience. This patient had been under the most eminent worm doctors in the metropolis, without benefit.

*Case* 2.—Miss T., aged 12, had suffered for three years from tænia, and taken turpentine, senna, calomel, and the usual drastic doses. The first dose of the kousso fully answered the purpose, for, although the actual termination of the worm was not obtained, the mass was traced to a fine point, slightly jagged, and the patient has been free from the symptoms of the disorder a very long time. A second dose was given in this case also; it acted gently, as the first had done, but the motion contained nothing remarkable. The price had now been reduced to four shillings.

It would be easy to multiply evidence on this subject, from the experience of my professional friends, but the two cases now brought forward are sufficient to prove that the Brayera anthelmintica deserves a place in our materia medica, and a constant moderate price is the only circumstance wanting to bring it into general use. It will be observed that its success did not depend upon the activity of its purgative quality. The action was gentle, and far less severe than that of the inefficacious drastic remedies which had preceded it; we must therefore conclude that its operation is specific, and, like all specifics, it will occasionally fail. There are many varieties of the tænia, and it is not improbable that to some of these it does not prove poisonous, if its failure does not arise from adulteration. It seems to be a harmless thing, of no striking property in the human body when the worm is absent. I have given two drachms, infused in hot water, to persons complaining of abdominal pains, who formerly suffered from tænia, and it has moved the bowels slightly, and relieved the pains, just as an ordinary dose of rhubarb would do, and with no other perceptible effect. It is now pretty generally known that the Brayera anthelmintica was discovered by the traveller Bruce; it is classed amongst the Boraceæ; and by De Candolle, in tribe v., Dryadeæ. It grows in Abyssinia to the height of twenty feet, and is cultivated everywhere for its anthelmintic properties. It is found in Tigre, Agame, and Shoa, and, according to Dr. Beke, through the entire table land of North Eastern Abyssinia. The bunches are gathered before the seeds are quite ripe, whilst still a number of florets remain unchanged. Its peculiar property resides chiefly in its bitter acrid resin, soluble in ether and in alcohol. The decoction strikes a dark green tint with a solution of the sesquichloride of iron. Dr. Pereira, from whose paper in the *Pharmaceutical Journal,* (Vol. x., No. 1,) much information on the subject may be gained, gives the following directions for its administration:—"The powdered flowers are to be mixed with luke-warm water, (for an adult, about ten ounces,) they are allowed to infuse for a quarter of an hour, a little lemon-juice is then to be swallowed, and the infusion being stirred up, the whole is taken, liquid and powder, at two or three draughts, at short intervals, being washed down by cold water and lemon-juice, To promote the operation, tea (without sugar or milk) may be taken. In three or four hours, if the remedy has not operated, a dose of castor oil, or a saline purgative should be administered."—p. 24.

Not unfrequently, both in public and private practice, patients present themselves suffering from tape-worm; sometimes very little inconvenience is complained of, but generally there are distinct symptoms of the disorder. I am inclined to think that the popular notion is well grounded, and that, till the head of the animal comes away, the patient is not cured. There is seldom more than one worm, and yet portions are constantly being separated, which, if they possessed independent vitality, and the power of reproduction, would assuredly fix themselves on some other portion of the intestinal tube.

The Abyssinians feed upon raw meat, and to this rude practice may be referred the prevalence of this malady amongst these people. The cysticercus is found in the flesh of the pig and sheep in our own country, and if the tænia be a developed cysticercus, its origin here would be accounted for. Some very humerous, and yet instructive remarks on this subject are contained in the last *Edinburgh Monthly Journal.* It is more than probable that the ova of the tænia, the ascaris, and some other parasitic animals, find their way into the human body through the unprepared or uncooked materials of our food. And it may be further remarked, that a low degree of vitality of the system greatly predisposes it to their attacks, and hence the value of steel, and other tonics, by way of prevention and restoration.—*Prov. Med. and Surg. Journ.*

